# Serum Conjugated Linoleic Acid and Risk of Incident Heart Failure in Older Men: The British Regional Heart Study

**DOI:** 10.1161/JAHA.117.006653

**Published:** 2018-01-06

**Authors:** S. Goya Wannamethee, Barbara J. Jefferis, Lucy Lennon, Olia Papacosta, Peter H. Whincup, Aroon D. Hingorani

**Affiliations:** ^1^ Department of Primary Care and Population Health University College London United Kingdom; ^2^ Population Health Research Institute St George's, University of London United Kingdom; ^3^ Institute of Cardiovascular Sciences University College London United Kingdom

**Keywords:** epidemiology, fatty acid, heart failure, Heart Failure, Diet and Nutrition, Aging

## Abstract

**Background:**

Evidence largely from animal studies suggests that conjugated linoleic acid (CLA) may have cardiovascular health benefits. However, few prospective studies have examined the association between CLA and cardiovascular disease. We have prospectively examined the association between serum CLA and incident coronary heart disease and heart failure (HF) in older men.

**Methods and Results:**

Prospective study of 3806 men, aged 60 to 79 years, without prevalent HF followed up for an average of 13 years, during which there were 295 incident HF cases. A high‐throughput serum nuclear magnetic resonance metabolomics platform was used to measure CLA concentration in serum, expressed as a percentage of total fatty acids (CLA%). CLA% was adversely associated with cholesterol and high‐density lipoprotein cholesterol but was inversely associated with C‐reactive protein and NT‐proBNP (N‐terminal pro‐B‐type natriuretic peptide; a marker of ventricular stress). No association was seen between CLA% and incident coronary heart disease. High CLA% was associated with significantly reduced risk of HF after adjustment for HF risk factors and C‐reactive protein (hazard ratio [95% confidence interval], 0.64 [0.43–0.96]; quartile 4 versus quartile 1). Elevated CLA% was associated with reduced HF risk only in those with higher dairy fat intake, a major dietary source of CLA (test for interaction *P*=0.03). The reduced risk of HF was partially explained by NT‐proBNP. High dairy fat intake was not associated with incident coronary heart disease but was associated with reduced risk of HF, largely because of the inverse effect of CLA.

**Conclusions:**

The finding that high CLA% is associated with lower risk of incident HF in older men requires confirmation in larger studies.

**Clinical Perspective**

**What Is New?**

- Conjugated linoleic acid (CLA) is a *trans* fatty acid that has been shown in clinical studies to have antiatherosclerotic, antidiabetic, anti‐inflammatory, and immune‐modulating properties.
- This is the first prospective study on the association between CLA and risk of incident heart failure (HF).
- CLA expressed as a percentage of total fatty acids was inversely associated with inflammation and NT‐proBNP (N‐terminal pro‐B‐type natriuretic peptide), a marker of neurohormonal activation and cardiac injury.
- High CLA expressed as a percentage of total fatty acids was associated with significantly reduced risk of incident HF (but not coronary heart disease) after adjustment for established risk factors for HF.

**What Are the Clinical Implications?**

- The novel finding of an inverse association between CLA expressed as a percentage of total fatty acids and HF requires confirmation in other populations.
- The observational data suggest that a diet rich in CLA may be protective against the development of HF and may have important implications for effective nutritional interventions towards the prevention of HF.
- Primary intervention trials in older people at risk of HF would be needed to confirm whether increasing CLA through supplements or diet would reduce risk of HF.

Conjugated linoleic acid (CLA) is a *trans* fatty acid (TFA) that naturally occurs as a mixture of positional and geometric isomers of LA. The amount of CLA in the human body seems to be directly related to the dietary intake of CLA.^1^ CLA is produced naturally in ruminant animals, such as cows, sheep, and goats. Dairy products are a primary source of *cis*‐9, *trans*‐11‐CLA (c9,t11‐CLA), the most prevalent CLA isomer, in the diet of humans.^1^, ^2^, ^3^ Epidemiological studies have shown a strong positive association between the intake of TFAs and coronary heart disease (CHD).^4^, ^5^ However, evidence largely based on animal models has paradoxically shown CLA to have antiatherosclerotic, antidiabetic, anti‐inflammatory, and immune‐modulating properties.^1^, ^2^, ^3^, ^6^, ^7^ In vitro and in vivo studies in rats have also shown CLA to suppress cardiac hypertrophy, a major risk factor for heart failure (HF).^8^ CLA has gained an increasing amount of attention in the past decade because of its potential health benefit^1^ and in the potential for naturally increasing the isomer c9,t11‐CLA content of milk and dairy products.^2^ However, in human studies, evidence on the antiatherosclerotic effects of CLA remains contradictory.^6^, ^7^, ^9^, ^10^, ^11^, ^12^, ^13^ There is a paucity of prospective studies on CLA and incident cardiovascular disease (CVD), but one study has shown higher CLA levels measured in adipose tissue to be associated with lower risk of myocardial infarction (MI).^14^ However, to date, no prospective studies have examined the association between CLA and incident HF. To further understand the cardiovascular health implications of CLA, we have examined the association between serum CLA and the following: (1) vascular risk factors, (2) incident CHD, and (3) incident HF in older men.

## Methods

The data, analytic methods, and study materials will not be made available to other researchers for purposes of reproducing the results or replicating the procedure.

The British Regional Heart Study is a prospective study involving 7735 men, aged 40 to 59 years, drawn from one general practice in each of 24 British towns, who were screened between 1978 and 1980.^15^ The population studied was socioeconomically representative of British men and comprises predominantly white Europeans (>99%). From 1998 to 2000, all surviving men, now aged 60 to 79 years, were invited for a 20th year follow‐up examination. Ethical approval has been obtained from all relevant local research ethics committees. All participants provided informed written consent to the investigation, which was performed in accordance with the Declaration of Helsinki. All men completed a mailed questionnaire, providing information on their lifestyle and medical history, had a physical examination, and provided a fasting blood sample. The samples were frozen and stored at −20°C on the day of collection and transferred in batches for storage at −70°C until analysis. Details of their medications were recorded at the examination. Twelve‐lead ECGs were recorded using a Siemens Sicard 460 instrument and were analyzed using Minnesota Coding definitions at the University of Glasgow ECG core laboratory. A total of 4252 men (77% of survivors) attended for examination. Blood measurements were available in 4049 men. We excluded 119 men with a history of HF, leaving 3930 men for analyses. The 1998 to 2000 examination served as the baseline for the current analyses.

### Cardiovascular Risk Factors

Details of measurement and classification methods for body mass index, smoking status, physical activity, social class, alcohol intake, blood pressure, blood lipids, plasma glucose, renal dysfunction, and forced expiratory volume in 1 second in this cohort have been described.^16^, ^17^, ^18^ Prevalent diabetes mellitus included men with a diagnosis of diabetes mellitus and men with a fasting blood glucose level ≥7 mmol/L. NT‐proBNP (N‐terminal pro‐B‐type natriuretic peptide) was determined using the Elecsys 2010 (Roche Diagnostics, Burgess Hill, UK).^17^ Troponin T was measured by a high‐sensitivity method on an e411 (Roche Diagnostics) using the manufacturer's calibrators and quality control material. C‐reactive protein (CRP) was assayed by ultrasensitive nephelometry (Dade Behring, Milton Keynes, UK). Electrocardiographic left ventricular hypertrophy was defined according to relevant Minnesota code (code 3.1 or 3.3). Atrial fibrillation was defined according to Minnesota codes 8.3.1 and 8.3.3.

### Dietary Assessment

Dietary intake was measured at baseline from 1998 to 2000 with a self‐administered food frequency questionnaire (FFQ) that was developed for use in the World Health Organization's Monitoring Trends and Determinants in Cardiovascular Disease Survey.^19^ Participants reported how frequently they usually ate 86 different food and drink items per week. Total macronutrient and micronutrient intakes of foods consumed were derived using a validated computer program to calculate the total nutrient composition of all foods reported as consumed in the FFQ. The FFQ used in this study has confirmed the known protective effect of the Mediterranean diet on CVD risk,^20^ providing evidence of the validity of the FFQ. On the basis of reported milk and butter intake, the men were divided into 4 groups of dairy fat intake: 1 (low), skimmed/semiskimmed milk and low‐fat cheese; 2, skimmed milk and full‐fat cheese or full‐fat milk and low‐fat cheese; 3, semiskimmed milk and full‐fat cheese; and 4 (high), full‐fat milk and high‐fat cheese.

### CLA and Other Fatty Acids

A high‐throughput serum nuclear magnetic resonance (NMR) metabolomics platform was used to quantify >200 metabolite measures from unthawed serum samples that represent a broad molecular signature of systemic metabolism.^21^ The metabolites were measured in a single experimental setup that allows for the simultaneous quantification of routine lipids, total lipid concentrations of 14 lipoprotein subclasses, fatty acid composition (including monounsaturated fatty acids [MUFAs], saturated fatty acids), and polyunsaturated fatty acids [PUFAs]), CLA, various glycolysis precursors, ketone bodies, and amino acids in absolute concentration units.^21^, ^22^ Applications of this high‐throughput metabolomics platform have previously been applied in both epidemiological and genetics studies^22^ and details of the experimentation have been described elsewhere.^21^, ^22^ The quantification of fatty acid biomarkers using the NMR approach has been validated by comparing NMR with gas chromatography in the Cardiovascular Risk in the Young Finn Study The fatty acids quantification was highly consistent between methods (*r*=0.92 for MUFA%, and *r*=0.94 for PUFA%), although CLA expressed as a percentage of total fatty acids (CLA%) was not specifically validated in this study.^23^ CLAs, PUFAs, saturated fatty acids, and MUFAs were expressed as a percentage of total fatty acids (CLA%, PUFA%, saturated fatty acid %, and MUFA%, respectively). CLA% was available in 3806 men.

### Follow‐Up

All men have been followed up from initial examination (1978–1980) to June 2012 for cardiovascular morbidity and mortality through general practitioners' medical records and the National Health Service Register for mortality. Follow‐up has been achieved for 99% of the cohort.^15^ Fatal CHD events were defined as death with CHD (*International Classification of Diseases, Ninth Revision* [*ICD‐9*] codes 410–414) as the underlying code. Evidence of nonfatal MI and HF was obtained by regular biennial reviews of participants' primary care records (including hospital and clinic correspondence) through to the end of the study period. A nonfatal MI was diagnosed according to World Health Organization criteria. Incident nonfatal HF was based on a physician diagnosis of HF recorded in primary care records and verified using details of available clinical information from primary and secondary care records (including symptoms, signs, investigations, and treatment response) to ensure consistency with current diagnostic practice^24^; any cases with a strong likelihood of alternative diagnoses were excluded from considerations. Fatal HF cases were those in which the diagnosis of HF was mentioned as the underlying cause of death (*ICD‐9* code 428). Incident HF included both incident nonfatal HF and fatal HF cases.

### Statistical Analyses

The men were divided into equal quartiles on the basis of the CLA% distribution. Tests for trend across the 4 CLA% groups were performed using the χ^2^ linear test for trend across the 4 groups for categorical variables and ANOVA for continuous variables. Cox proportional hazards model was used to assess the multivariate‐adjusted hazard ratios (relative risk) by quartiles of CLA%. In multivariate analyses, smoking (never, long‐term ex‐smokers [>15 years], recent ex‐smokers [<15 years], and current smokers), social class (manual versus nonmanual), physical activity (4 groups), prevalent diabetes mellitus (yes/no), prevalent MI (yes/no), use of antihypertensive treatment (yes/no), left ventricular hypertrophy (yes/no), renal dysfunction (yes/no), and atrial fibrillation (yes/no) were fitted as categorical variables. Systolic blood pressure, body mass index, high‐density lipoprotein cholesterol, estimated glomerular filtration rate, CRP, PUFA%, and NT‐proBNP were fitted continuously. Subsidiary analysis was stratified by men with and without previous MI. Analyses were also performed by stratifying the men by high and low NT‐proBNP level and by dairy fat intake groups. Test for interaction was performed by including an interaction term, CLA%×dairy fat intake groups (NT‐proBNP groups), with CLA% fitted continuously.

## Results

During the mean follow‐up of 13 years from 1998 to 2000 to July 2012, there were 295 incident HF cases and 413 major CHD events in the 3806 men with no diagnosed HF. The mean (SD) CLA% level in this study population was 0.38 (0.17).

### CLA and Vascular Risk Factors

Table 1 shows baseline characteristics in the study population by quartiles of CLA%. Those with high circulating CLA% tended to be older, more inactive, and heavier drinkers and had a higher body mass index, but they showed a lower prevalence of MI, atrial fibrillation, and use of antihypertensive treatment than those with lower CLA%. CLA% related to saturated dairy fat intake and frequency of beef and lamb intake and was significantly associated with increased dietary total fat (g/d) and saturated fat intake (g/d). CLA% related positively to saturated fatty acid % and MUFA% but inversely to PUFA%. Elevated CLA% was associated with lower high‐density lipoprotein cholesterol, higher total cholesterol, and higher systolic blood pressure but was inversely associated with CRP and NT‐proBNP (but not cardiac troponin T). CLA% remained adversely associated with blood lipids and blood pressure and inversely associated with CRP and NT‐proBNP after adjustment for age and body mass index and on exclusion of men with prevalent MI (Table 2).

**Table 1:** Baseline Characteristics by Quartiles of Serum CLA% in 3890 Men Without HF

Characteristics	CLA% (Quartiles)				*P* Value for Trend
	1	2	3	4	
	(n=915)	(n=960)	(n=971)	(n=960)	
	(<0.26)	(0.26–0.35)	(0.36–0.46)	(≥0.47)	
Age, y	68.5	68.4	68.7	69.0	0.04
BMI, kg/m^2^	26.3	27.0	27.0	27.0	<0.0001
Smokers, %	12.5	11.9	12.0	14.4	0.22
Manual, %	56.2	55.7	50.3	52.0	0.01
Inactive, %	32.3	32.2	34.8	36.7	0.02
Heavy drinkers, %	2.9	3.8	3.5	4.9	0.04
Myocardial infarction, %	10.4	12.2	9.2	8.1	0.02
Diabetes mellitus, %	6.7	7.0	9.2	8.1	0.56
LVH, %	8.7	7.6	6.9	7.3	0.48
AF, %	4.6	3.3	3.6	2.1	0.005
Renal dysfunction, %	14.4	14.8	17.2	14.9	0.45
Use of antihypertensive drugs, %	33.5	35.4	31.5	29.8	0.02
Dietary intake (FFQ)					
Beef >2 times/wk, %	32.9	37.6	41.0	44.1	<0.0001
Lamb >2 times/wk, %	7.5	8.9	11.7	16.2	<0.0001
High dairy fat, %	7.4	13.4	16.4	21.6	<0.0001
Total fat, g/d	66.9±23.9	68.7±25.1	72.0±25.7	79.8±28.2	<0.0001
Total energy, kcal	2056±495	2076±522	2130±533	2200±542	<0.0001
Serum fatty acids					
PUFA%	39.0 (3.30)	37.2 (3.33)	36.1 (3.23)	37.8 (3.62)	<0.0001
MUFA%	24.5 (3.25)	26.1 (3.31)	27.0 (3.25)	28.7 (3.49)	<0.0001
SFA%	36.5 (1.61)	36.7 (1.57)	36.9 (1.49)	37.6 (1.82)	<0.0001
Biological factors					
SBP, mm Hg	148.2 (23.8)	148.3 (24.0)	150.2 (24.2)	150.8 (23.7)	0.005
Cholesterol, mmol/L	5.73 (0.98)	6.00 (1.05)	6.10 (1.09)	6.25 (1.11)	<0.0001
HDL‐C, mmol/L	1.36 (0.35)	1.33 (0.35)	1.31 (0.32)	1.29 (0.34)	<0.0001
Glucose, mmol/L^a^	5.81 (5.22–6.04)	5.87 (5.26–6.12)	5.81 (5.25–6.06)	5.93 (5.32–6.19)	0.05
FEV_1_, L	2.59 (0.69)	2.64 (0.65)	2.63 (0.65)	2.56 (0.64)	0.21
CRP, mg/L^a^	1.84 (0.80–3.93)	1.67 (0.82–3.12)	1.67 (0.81–3.38)	1.68 (0.83–3.35)	0.08
cTnT, pg/mL^a^	11.94 (8.90–15.80)	11.58 (8.60–15.70)	11.94 (8.70–15.70)	12.06 (9.0–16.5)	0.34
NT‐proBNP, pg/mL^a^	104.6 (47–194)	100.5 (49–199)	94.6 (45–190)	89.2 (43–178)	0.002

Data are given as mean (SD unless otherwise specified. AF indicates atrial fibrillation; BMI, body mass index; CLA%, conjugated linoleic acid expressed as a percentage of total fatty acids; CRP, C‐reactive protein; cTnT, cardiac troponin T; FEV_1_, forced expiratory volume in 1 second; FFQ, food frequency questionnaire; HDL‐C, high‐density lipoprotein cholesterol; HF, heart failure; LVH, left ventricular hypertrophy; MUFA%, monounsaturated fatty acid expressed as a percentage of total fatty acids; NT‐proBNP, N‐terminal pro‐B‐type natriuretic peptide; PUFA%, polyunsaturated fatty acid expressed as a percentage of total fatty acids; SBP, systolic blood pressure; and SFA%, saturated fatty acid expressed as a percentage of total fatty acids.

a Data are given as geometric mean (interquartile range).

**Table 2:** Spearman Partial Correlation Coefficients Between Serum CLA% and Biological Markers in All Men With No Prevalent HF

Variable	Age Adjusted	Age+BMI Adjusted	Age+BMI Adjusted^a^
SBP	0.04^b^	0.04^b^	0.03^b^
Cholesterol	0.19^b^	0.20^b^	0.19^b^
HDL cholesterol	−0.09^b^	−0.06^b^	−0.07^b^
Glucose^c^	0.05^b^	0.04^b^	0.04^b^
FEV_1_	−0.01	−0.01	−*0.01*
CRP^c^	−0.04^b^	−0.06^b^	−0.05^b^
cTnT^c^	0.007	−0.007	−0.002
NT‐proBNP^c^	−0.08^b^	−0.06^b^	−0.08^b^

BMI indicates body mass index; CLA%, conjugated linoleic acid expressed as a percentage of total fatty acids; CRP, C‐reactive protein; cTnT, cardiac troponin T; FEV_1_, forced expiratory volume in 1 second; HDL, high‐density lipoprotein; HF, heart failure; NT‐proBNP, N‐terminal pro‐B‐type natriuretic peptide; and SBP, systolic blood pressure.

a Excludes 381 men with prevalent myocardial infarction.

b *P*<0.05.

c Logarithm.

### CLA, Dairy Fat Intake, and Major CHD

CLA% did not relate to major CHD events. The age‐adjusted risks of CHD in all men for the 4 quartiles of CLA% were 1.00, 0.95 (0.72–1.26), 0.93 (0.71–1.24), and 1.13 (0.86–1.47). Similarly, dairy fat intake was not associated with major CHD events. The age‐adjusted relative risks were 1.00, 0.90 (0.59–1.38), 0.94 (0.71–1.25), and 1.06 (0.77–1.46). Exclusion of men with MI made little difference to these findings.

### CLA, Dairy Fat Intake, and Incident HF

CLA% was inversely associated with HF after adjustment for CVD risk factors and PUFA (model 2; Table 3). However, further adjustment for NT‐proBNP attenuated the association. The reduced risk of HF associated with elevated CLA% was seen in both men with and without MI (Table 3). When stratified by levels of NT‐proBNP, the reduced risk of HF associated with elevated CLA% was seen in both men with high (≥188 pg/mL; top quartile) levels of NT‐proBNP and those with levels <188 pg/mL. The adjusted hazard ratios (95% confidence interval) (top quartile versus bottom quartile) were 0.67 (0.42–1.09) and 0.59 (0.35–1.01), respectively. There was no evidence of an interaction between CLA% and NT‐proBNP with HF risk (*P*=0.81).

**Table 3:** Incidence Rate/1000 Person‐Years and Adjusted Relative HRs and 95% CIs for Incident HF by Quartiles of CLA% in Men With No Prevalent HF

Variable	CLA% (Quartiles)			
	1	2	3	4
All men				
N	915	960	971	960
Rate/1000 person‐years (n)	8.1 (86)	7.7 (79)	7.5 (77)	5.5 (56)
HR (95% CI)				
Age adjusted	1.00	0.91 (0.67–1.24)	0.88 (0.65–1.20)	0.61 (0.44–0.86)
Model 1	1.00	0.88 (0.65–1.20)	0.95 (0.70–1.31)	0.63 (0.44–0.89)
Model 2	1.00	0.89 (0.64–1.23)	0.97 (0.69–1.35)	0.64 (0.43–0.96)
Model 3	1.00	0.89 (0.64–1.24)	1.00 (0.71–1.41)	0.70 (0.47–1.05)
No MI				
N	818	843	882	882
Rate/1000 person‐years (n)	7.1 (69)	6.0 (56)	6.0 (57)	5.0 (47)
HR (95% CI)				
Age adjusted	1.00	0.81 (0.87–1.16)	0.80 (0.56–1.14)	0.63 (0.43–0.91)
Model 1	1.00	0.82 (0.57–1.18)	0.82 (0.57–1.18)	0.67 (0.46–0.98)
Model 2	1.00	0.80 (0.55–1.16)	0.80 (0.54–1.16)	0.63 (0.41–0.98)
Model 3	1.00	0.76 (0.51–1.11)	0.83 (0.56–1.23)	0.65 (0.41–1.01)
With MI				
N	97	117	89	78
Rate/1000 person‐years (n)	18.4 (17)	23.4 (23)	25.6 (20)	12.4 (9)
HR (95% CI)				
Age adjusted	1.00	1.22 (0.64–2.31)	1.34 (0.69–2.59)	0.63 (0.28–1.43)
Model 1	1.00	0.94 (0.47–1.87)	1.39 (0.67–2.80)	0.46 (0.18–1.15)
Model 2	1.00	1.08 (0.53–2.21)	1.76 (0.83–3.75)	0.61 (0.23–1.66)
Model 3	1.00	1.10 (0.52–2.31)	1.64 (0.76–3.67)	0.64 (0.23–1.75)

Model 1, adjusted for age, smoking, heavy drinking, social class, body mass index, high‐density lipoprotein cholesterol, systolic blood pressure, atrial fibrillation, left ventricular hypertrophy, prevalent diabetes mellitus, prevalent myocardial infarction, and C‐reactive protein. Model 2, adjusted for model 1 plus polyunsaturated fatty acids. Model 3, adjusted for model 2 plus N‐terminal pro‐B‐type natriuretic peptide. CI indicates confidence interval; CLA%, conjugated linoleic acid expressed as a percentage of total fatty acids; HF, heart failure; HR, hazard ratio; and MI, myocardial infarction.

High dietary dairy fat intake was strongly associated with having elevated CLA% (Table 4) and was also associated with reduced risk of HF, even after exclusion of men with MI. However, adjustment for CLA% attenuated the association (Table 4). We also examined the relationship between CLA% and risk of HF stratified by dietary dairy fat intake. Because of the smaller numbers in the lower dietary dairy intake groups, we combined the 2 lowest dairy intake groups together to achieve sufficient numbers in the stratified analysis. The lower risk of HF associated with elevated CLA% was only evident in those with higher levels of dairy fat intake (Table 5). No benefit was seen in those with lower dairy intake, and a test for interaction between CLA% and dairy fat intake and risk of HF was significant (*P*=0.03). By contrast, no interaction was seen with frequency of beef intake (>2/week versus ≤2/week) (*P*=0.51).

**Table 4:** Incidence Rate/1000 Person‐Years and Adjusted Relative HRs and 95% CIs for Incident HF According to Dietary Dairy Fat Intake in Men With No Prevalent HF

Variable	Dairy Fat Intake			
	1 (Low)	2	3	4 (High)
All men	549	363	2014	806
CLA%, mean±SD	0.32±0.15	0.33±0.17	0.38±0.16	0.45±0.19
Rate/1000 person‐years	9.8	6.8	7.2	6.0
HR (95% CI)				
Age adjusted	1.00	0.77 (0.49–1.21)	0.73 (0.54–0.99)	0.54 (0.37–0.79)
Model 1	1.00	0.87 (0.54–1.38)	0.88 (0.64–1.21)	0.71 (0.47–1.06)
Model+CLA%	1.00	0.88 (0.55–1.40)	0.92 (0.67–1.27)	0.78 (0.52–1.19)
No MI				
N	452	320	1822	765
Rate/1000 person‐years	8.4	5.4	5.8	5.6
HR (95% CI)				
Age adjusted	1.00	0.71 (0.41–1.21)	0.69 (0.48–0.91)	0.58 (0.38–0.87)
Model 1	1.00	0.74 (0.43–1.28)	0.75 (0.52–1.08)	0.63 (0.40–0.98)
Model 1+CLA%	1.00	0.83 (0.53–1.32)	0.84 (0.62–1.15)	0.68 (0.45–1.10)

Model 1, adjusted for age, smoking, heavy drinking, body mass index, high‐density lipoprotein cholesterol, systolic blood pressure, atrial fibrillation, left ventricular hypertrophy, prevalent diabetes mellitus, prevalent myocardial infarction, and C‐reactive protein. 2, skimmed mik and full fat cheese or full fat milk and low fat cheese. 3, semi‐skimmed milk and full fat cheese. 4, full‐fat milk and high‐fat cheese. Low, skimmed/semiskimmed milk and low‐fat cheese. CI indicates confidence interval; CLA%, serum conjugated linoleic acid expressed as a percentage of total fatty acids; HF, heart failure; HR, hazard ratio; and MI, myocardial infarction.

**Table 5:** Incidence Rate/1000 Person‐Years and Adjusted Relative HRs and 95% CIs for Incident HF by Quartiles of CLA% According to Dietary Dairy Fat Intake in Men With No Prevalent HF

Variable	CLA% (Quartiles)			
	1 (Low)	2	3	4 (High)
Lower dairy intake				
N (n)	316 (26)	258 (26)	210 (25)	128 (16)
Rate/1000 person‐years	7.6	9.1	7.2	12.3
HR (95% CI)				
Age adjusted	1.00	1.23 (0.71–2.13)	0.93 (0.50–1.73)	1.60 (0.87–2.95)
Model 1	1.00	1.24 (0.69–2.21)	0.98 (0.52–1.86)	1.53 (0.80–2.91)
Model 1+NT‐proBNP	1.00	1.23 (0.69–2.21)	1.05 (0.54–2.04)	1.67 (0.87–3.21)
Medium dairy intake				
N (n)	470 (43)	514 (49)	543 (44)	765 (26)
Rate/1000 person‐years	8.6	7.5	7.5	5.0
HR (95% CI)				
Age adjusted	1.00	0.90 (0.59–1.37)	0.90 (0.59–1.57)	0.55 (0.34–0.90)
Model 1	1.00	0.81 (0.53–1.26)	0.93 (0.61–1.43)	0.49 (0.29–0.81)
Model 1+NT‐proBNP	1.00	0.85 (0.54–1.33)	0.94 (0.60–1.46)	0.51 (0.30–0.87)
High dairy intake				
N (n)	100 (10)	169 (10)	206 (17)	331 (13)
Rate/1000 person‐years	9.6	5.7	8.2	3.7
HR (95% CI)				
Age adjusted	1.00	0.61 (0.26–1.48)	0.92 (0.42–2.01)	0.43 (0.19–0.98)
Model 1	1.00	0.53 (0.21–1.32)	0.86 (0.38–1.99)	0.41 (0.17–0.98)
Model 1+NT‐proBNP	1.00	0.48 (0.19–1.24)	0.99 (0.42–2.55)	0.49 (0.20–1.20)

Model 1, adjusted for age, smoking, heavy drinking, body mass index, high‐density lipoprotein cholesterol, systolic blood pressure, atrial fibrillation, left ventricular hypertrophy, prevalent diabetes mellitus, prevalent myocardial infarction, and C‐reactive protein. High, high‐fat milk and full‐fat cheese. Medium, semiskimmed milk and full‐fat cheese or full‐fat milk and low‐fat cheese. Lower (groups 1 and 2), skimmed/semiskimmed milk and low‐fat cheese or skimmed milk and full‐fat cheese. CI indicates confidence interval; CLA%, serum conjugated linoleic acid expressed as a percentage of total fatty acids; HF, heart failure; HR, hazard ratio; and NT‐proBNP, N‐terminal pro‐B‐type natriuretic peptide.

## Discussion

In this study of older British men, there was no association between circulating levels of CLA, relative to total fatty acids, and incident MI, despite its adverse effects on blood lipids. CLA% was, however, shown to be associated with a lower risk of HF, which was independent of potential confounders and inflammatory markers. The relationship between CLA% and incident HF was, to some extent, mediated by NT‐proBNP, a marker of cardiac damage and ventricular stress, and was only seen in those with higher dairy fat intake. Our study adds to the growing literature on CLA and CVD further by examining the association between circulating CLA% and a wide range of vascular risk markers, including NT‐proBNP and incident HF, not previously examined. Dietary dairy fat intake, which was significantly associated with CLA%, was not associated with incident MI, a finding that is consistent with numerous other studies.^25^ However, it was associated with a lower risk of HF, which was largely accounted for by CLA%.

### CLA and CVD Risk

CLAs are a collection of positional and geometric isomers of linoleic acid, with the c9,t11‐CLA and *trans*‐10, *cis*‐12‐CLA being the 2 main isomers known to possess biological activity.^3^ Although numerous animal studies have shown CLA to exert antiatherogenic properties,^1^, ^2^, ^3^, ^7^ the beneficial effects of CLA on CVD risk in humans are less conclusive.^7^ Many studies have shown unfavorable effects of CLA on lipoprotein levels, similar to that of other TFAs,^6^ as was observed in this study, whereas others have shown beneficial or no effects.^7^, ^9^ We have observed a positive association between CLA% and blood pressure, in contrast to studies suggesting that CLA supplementation may decrease blood pressure.^3^ However, CLA% was associated with lower levels of CRP, consistent with clinical and dietary intervention studies suggesting an anti‐inflammatory effect of CLA.^2^, ^3^, ^13^ Other studies, however, have reported no association or positive associations between CLA and inflammation.^11^, ^12^ The c9,t11‐CLA isomer is the principal dietary form of CLA, accounting for as much as 85% to 90% of total CLA in dairy products, and is the predominant isomer present in human plasma.^26^ The *trans*‐10, *cis*‐12‐CLA isomer is not produced in detectable amounts naturally but is found in larger amounts as a product of commercial CLA synthesis. Many of the studies on the effects of CLA on cardiovascular risk factors have focused on supplementary CLA, which commonly consists of roughly equal portions of c9,t11‐CLA and *trans*‐10, *cis*‐12‐CLA; it is possible that the 2 isomers may have differing effects on cardiovascular risk. This may account for the inconsistencies seen between studies on the effects of CLA on cardiovascular risk factors.

### CLA and HF Risk

A novel finding in this study is the inverse association seen between serum CLA% and risk of incident HF, which was not explained by established risk factors for HF. A possible mechanism may involve inhibition of cardiac hypertrophy.^8^ Animal and clinical studies have shown CLA to suppress cardiomyocyte hypertrophy through activation of the peroxisome proliferator activated receptor isoforms,^8^ which play an important role in cardiac function and fatty acid use in the healthy heart.^27^ Cardiac hypertrophy occurs in response to aberrant stress signals (eg, neurohormonal activation, inflammation, or cardiac injury). The findings that CLA% was inversely associated with NT‐proBNP, a marker of neurohormonal activation and cardiac injury, are consistent with the suggestion that CLA inhibits cardiac hypertrophy.^8^ The association between CLA% and incident HF was mediated by NT‐proBNP, suggesting that CLA may prevent the development of cardiac hypertrophy, a major factor leading to HF.

Although circulating c9,t11‐CLA in plasma correlates strongly with dietary intake of c9,t,11‐CLA,^28^ CLA in humans may not be totally determined by dietary CLA because CLA may also reflect metabolism. There is evidence of conversion to CLA of TFAs in humans.^29^ Vaccenic acid (VA), a major TFA in milk fat, has been shown to be converted to rumenic acid, an isomer of CLA, in human tissues by the Δ‐9‐desaturase enzyme, with an average of 19% of dietary VA being converted to c9,t11‐CLA.^29^ VA is the only known dietary precursor of c9,t11‐CLA. We did not have measures of VA. However, VA has not been shown to be associated with lower risk of HF in those without MI,^30^ whereas CLA was shown to be associated with reduced HF, even in men without MI. The finding that only elevated CLA% in men with higher dietary dairy fat intake (groups 3 and 4) was associated with reduced risk of HF suggests that CLA derived from dairy products and not from fatty acid metabolism is protective. Moreover, the difference in findings between elevated CLA% and HF risk in those with lower and those with higher dairy fat intake suggests that it is the c9,t11‐ CLA isomer that accounts for >75% of CLA in milk; this is the protective isomer, rather than the t10,c12‐CLA isomer, which accounts for <1% of CLA in milk.^26^, ^31^

The biological plausibility and inhibition of cardiac hypertrophy shown in animal studies and the inverse association seen with NT‐proBNP in this study, a marker of cardiac hypertrophy, suggest that CLA derived from dairy products may specifically be involved in the development of HF. Although many trials of the effects of CLA supplements on CVD risk factors have been disappointing, most have focused on overall traditional CVD or CHD risk factors, such as lipids and blood glucose, rather than changes in left ventricular structure and function.

### CLA and Incident CHD

In contrast to HF, we observed no association between CLA% and development of CHD in these older men. However, one case‐control study conducted in a Costa Rican population (average age, 58 years) showed c9,t11‐CLA in adipose tissue to be associated with lower risk of MI.^14^ Because c9,t11‐CLA is the predominant isomer present in human plasma, the difference in findings may be because of the different assay methods used or the different characteristics of the study group.

### Strengths and Limitations

Strengths and limitations of the study require consideration. This is the first study to examine the association between circulating CLA% and incident HF in a general population of older men who constitute a high‐risk group for CVD and HF. The study population is socially representative of the UK population, and follow‐up rates in the British Regional Heart Study are exceptionally high.^15^ However, it was based on an older predominantly white male population of European extraction, so that the results cannot be generalized directly to women, younger populations, or other ethnic groups. The current findings are based on physician‐diagnosed HF, which has yielded estimates of HF incidence consistent with those of earlier reports.^32^, ^33^ Moreover, the determinants of HF in this study population (including obesity, NT‐proBNP, lung function, and heavy drinking)^16^, ^17^, ^18^, ^34^ generally accord with prior data and suggest that the HF outcome used was valid. However, echocardiographic measurements were not routinely performed, and we were not able to differentiate systolic and diastolic HF. Dietary intake was assessed using an FFQ, which has previously been validated against weighed food intakes and serum levels in British populations,^35^, ^36^ and the dietary intake of participants was broadly comparable with those from the National Diet and Nutrition Survey.^37^ However, FFQs are more prone to measurement error compared with some other dietary measures, and in older populations, nonresponse to FFQ questions could have increased the chance of dietary underreporting. The present study was based on a prospective observational study and was not a randomized trial. We were not able to differentiate the different CLA isomers, and we had no measure of VA. CLA was measured using a high‐spectroscopy NMR platform and not the standard gas chromatography method for measuring fatty acids. However, fatty acids composition quantified by NMR from fasting serum samples has been shown to correlate highly with measures using gas chromatography (correlation coefficient ranging from 0.92 to 0.94),^23^ suggesting that the method is accurate.

## Conclusion

This study may have important implications for effective nutritional intervention towards the prevention of HF in older men who are at high risk of developing HF. This is the first study on the association between CLA% and risk of HF, and our findings need confirmation in other populations. If so, primary intervention trials in older people at risk of HF are needed to confirm whether increasing CLA through supplements or diet would reduce risk of HF.

## Independent Data Access and Analysis

Wannamethee had full access to all the data in the study and takes responsibility for their integrity and the data analysis.

## Sources of Funding

The British Regional Heart Study is a Research Group supported by the British Heart Foundation (BHF) Programme grant (RG/13/16/30528). Metabolite measurements were funded by a special projects BHF grant (SP/13/6/30554).

## Disclosures

None.
